# Head and Neck Non-Melanoma Skin Cancer Treated By Superficial X-Ray Therapy: An Analysis of 1021 Cases

**DOI:** 10.1371/journal.pone.0156544

**Published:** 2016-07-01

**Authors:** Daniel Grossi Marconi, Bruno da Costa Resende, Erick Rauber, Paula de Cassia Soares, Jose Maria Fernandes, Niraj Mehta, Andre Lopes Carvalho, Patrick A Kupelian, Allen Chen

**Affiliations:** 1 Barretos Cancer Hospital, Department of Radiation Oncology, Barretos, Brazil; 2 South Florida Radiation Oncology, Miami, Florida, United States of America; 3 Barretos Cancer Hospital, Head and Neck Division, Barretos, Brazil; 4 Department of Radiation Oncology, University of California Los Angeles, Los Angeles, California, United States of America; University of California Davis, UNITED STATES

## Abstract

**Introduction:**

To report a single-institutional experience with the use of Superficial X-Ray Therapy (SXRT) for head and neck non-melanoma skin cancer (N-MSC) and to compare outcomes by prescribed fractionation schedules.

**Materials and Methods:**

The medical records of 597 patients with 1021 lesions (720 BCC, 242 SCC, 59 SCC in situ) treated with kilovoltage radiation from 1979–2013 were retrospectively reviewed. The majority of patients were treated according to 1 of 3 institutional protocols based on the discretion of the radiation oncologist: 1) 22 x 2.5 Gy; 2) 20 x 2.5 Gy; 3) 30 x 2.0 Gy. "T" stage at first presentation was as follows: Tis (59); T1 (765); T2 (175); T3 (6), T4 (9); Tx, (7). All patients were clinical N0 and M0 at presentation. Chi-square test was used to evaluate any potential association between variables. The Kaplan-Meier method was used to analyze survival with the Log Rank test used for comparison. A Cox Regression analysis was performed for multivariate analysis.

**Results:**

The median follow up was 44 months. No significant difference was observed among the 3 prescribed fractionation schemes (p = 0.78) in terms of RTOG toxicity. There were no failures among SCC in situ, 37 local failures (23 BCC, 14 SCC), 5 regional failures (all SCC) and 2 distant failures (both SCC). For BCC, the 5-year LC was 96% and the 10-year LC was 94%. For SCC the corresponding rates of local control were 92% and 87%, respectively (p = 0.03). The use of >2.0 Gy daily was significantly associated with improved LC on multivariate analysis (HR: 0.17; CI 95%: 0.05–0.59).

**Conclusion:**

SXRT for N-MSC of the head and neck is well tolerated, achieves excellent local control, and should continue to be recommended in the management of this disease. Fractionation schedules using >2.0 Gy daily appear to be associated with improved LC.

## Introduction

Radiotherapy is an effective primary treatment for non-melanoma skin cancer (N-MSC), with cure rates in most series exceeding 90%, generally equivalent to surgery [1–6]. It is also appropriate for lesions that have recurred after previous resection [1]. Particularly, this treatment is appropriate in the head and neck region, especially for the nose, eyelids and ears, because it produces equivalent cure rates to surgery and can yield superior cosmetic outcomes [7,8]. Despite these characteristics, the use of Superficial X-Ray Therapy (SXRT) has declined in recent years due to a myriad of reasons, including the technical improvement of Mohs micrographic surgery [6], [8–9].

The objective is to report a single-institutional experience with the use of SXRT for head and neck N-MSC (basal cell carcinoma—BCC, squamous cell carcinoma—SCC, and non-invasive squamous cell carcinoma—SCC *in situ*) and to compare outcomes by prescribed fractionation.

## Material And Methods

### Patients

The Barretos Cancer Hospital IRB approved this study. All information was anonymized and de-identified prior to analysis.

We conducted a direct chart analysis of 597 patients with histological diagnosis of N-MSC treated with SXRT in an oncology hospital (Barretos Cancer Hospital) from 2000 to 2005. Every lesion was considered as an independent cancer and some metachronic lesions in the same patient were included in our study even before to 2000 or after 2005, in such a way that lesions treated from 1979 to 2013 were included in the analysis. In an attempt to standardize the classification all lesions were re-staged using the AJCC 7th edition reference. No patients had clinical evidence of regional lymph node or distant disease at presentation. No patient had underlining immunocompromised status.

### Treatment

All lesions were treated using the same machine: a Stabilipan II (Siemens, Erlanger, Germany), consisting of energies ranging from 80–200 keV. Energy was selected based on tumor characteristics. The most commonly used energy was 80 keV with a filter of 2 mm aluminum. When using energies of 140 KeV and 200 KeV, the filters were 0.5 mm copper and 10 mm copper, respectively.

By definition, Grenz rays use tube voltages of 5–20 kV, soft x-rays with 20–100 KV, superficial x-rays with 60–100 kV, and orthovoltage x-rays with 200–400 kV. The term SXRT used in this series referred to the usage of both superficial x-ray therapy and orthovoltage.

The majority of lesions (877 = 86%) were treated according to 1 of 3 institutional protocols based on the discretion of the radiation oncologist: 1) 22 x 2.5 Gy (55 Gy total); 2) 20 x 2.5 Gy (50 Gy total); 3) 30 x 2.0 Gy (60 Gy total), by the discretion of the physician. The remaining 144 treatments (14%) with other fractionation schemes were as follows: 1 = 28x1.8 Gy; 79 = 22-37x2 Gy; 21 = 20-25x2.4 Gy; 37 = 21-24x2.5 Gy; and 6 = 15-17x3 Gy. Patients and treatment characteristics are shown in Table 1.

**Table 1:** Tumor and Patients Characteristics.

Variables	Categories
**Age**	< 50: 197
	50–70:521
	> 70: 303
**Gender**	Male: 498
	Female: 523
**Race**	Caucasian: 1000
	African: 20
	Asian: 1
**T stage**	Tis—59
	T1—765
	T2—175
	T3—6
	T4—9
	Tx—7
**Histology**	BCC—720
	SCC—242
	SCC in situ—59
**Indication**	Primary—939
	Adjuvant—65
	Salvage—17
**Fractionation (schemes)**	20 x 2.5 Gy−273
	22 x 2.5 Gy−185
	30 x 2 Gy−419
	Other—144
**Fractionation (fraction size)**	2 Gy−500
	>2Gy−521
**Energy (KeV)**	80–909
	140–73
	200–39

Lead eye shielding was regularly performed while treating regions with potential excessive dose to this structure.

In planning, all lesions were drawn (GTV) and the radiation field was determined by institutional protocol with a margin of at least 0.5 to 1 cm circumferentially. The fractions were administered every day, 5 times per week, for 4–6 weeks.

### Data and Statistical Analysis

Local control (LC) was defined by the time between the end of treatment and the first evidence of local disease progression. The appearance of any biopsy-proven lesion with the same histology in a prior treatment field was considered a treatment failure.

Analysis of PFS was not calculated due to the small number of events.

In the event of regional or systemic progression, it was identified the primary lesion through the following arbitrary criteria, in descending order of importance: 1) Histology compatible; 2) Location compatible; 3) Staging "T" greatest; 4) Injury older. Thus, the remaining lesions were considered "controlled" and only the primary lesion was considered for local, regional or distant progression.

Per protocol, follow up consultation is performed every 6 months in the first 2 years and yearly thereafter. Toxicity was described as per RTOG classification.

For the calculations of LC lesions classified as SCC *in situ* were excluded.

The follow up was defined as time between the end of treatment to either the event or the most recently dated medical record.

The chi-square test was used to evaluate potential association between variables. The Kaplan-Meier method was used to analyze survival with the Log Rank test used for comparison. A Cox Regression analysis was performed for the multivariate analysis. We used a statistically significant a p-value of less than 0.05 for all analyses, using the software SPSS v.22.

## Results

**Fig 1:**
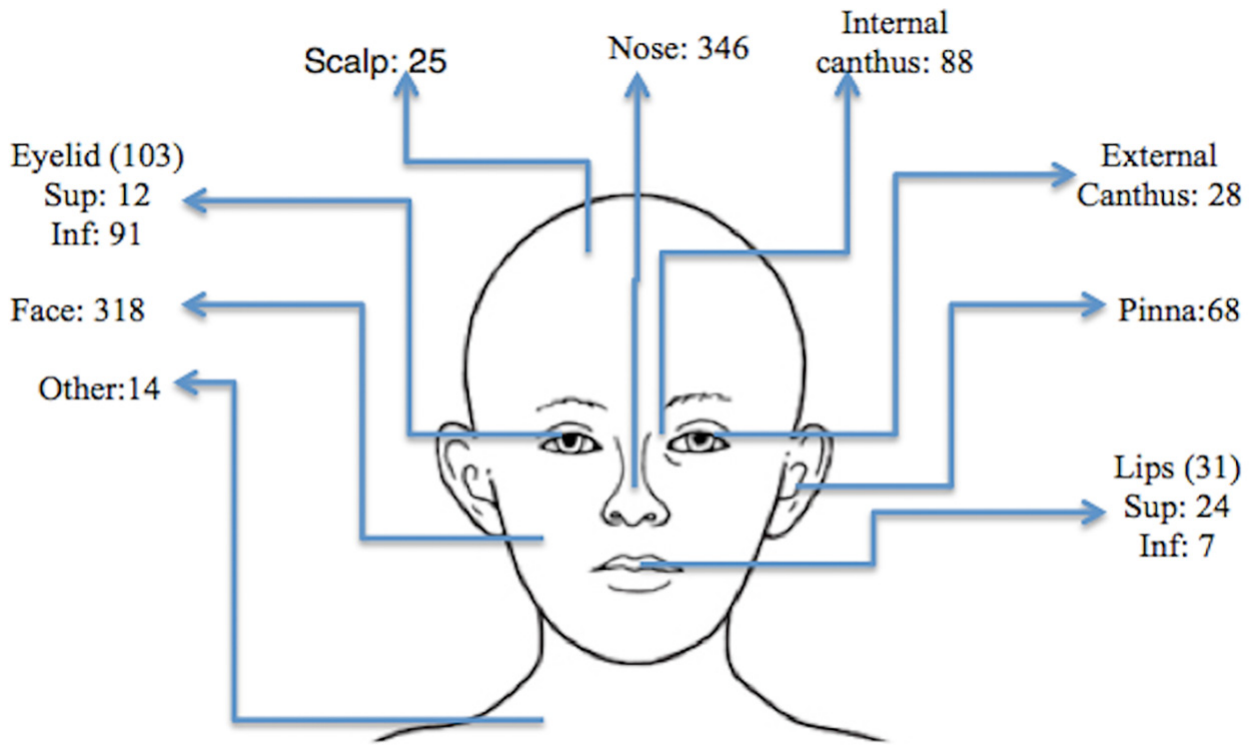
Anatomic distribution of lesions.

A total of 1021 N-MSC (720 BCC, 242 SCC, 59 SCC *in situ*) involving the head and neck treated with SXRT from 1979 to 2013 were retrospectively reviewed. Fig 1 demonstrates the anatomic location of lesions and Table 1 presents patient and disease characteristics.

The median follow up was 44 months (range: 1–406 months). Among the 17 salvage treatments, there was one local failure and one patient with both regional and distant failure. In the primary/adjuvant group, there were 36 local failures (23 BCC, 13 SCC), 4 regional failures (all SCC) and 1 distant failure (SCC)–in the lungs. There were no recurrences among the *in situ* lesions.

**Fig 2:**
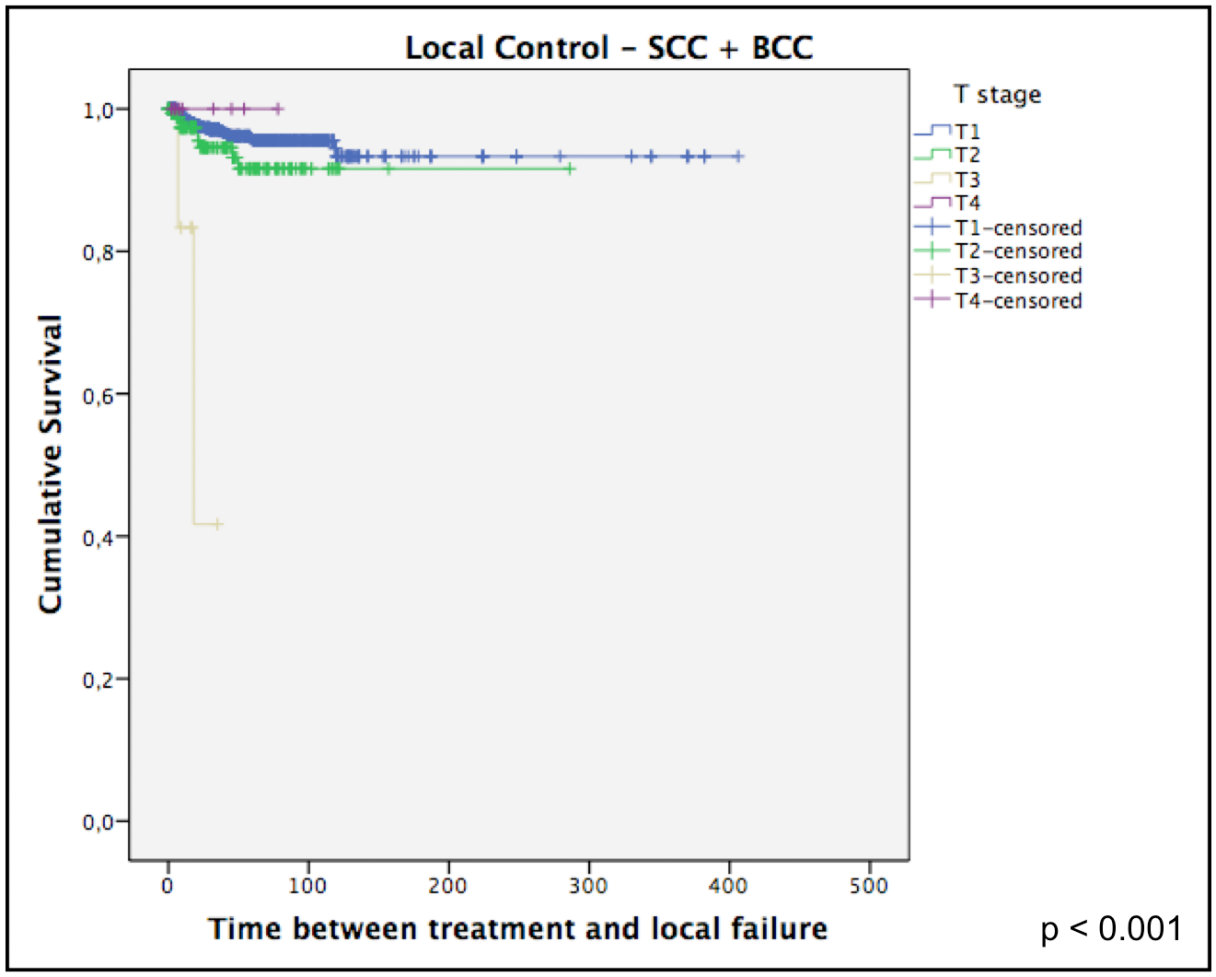
Local control only for BCC.

When looking only to BCC, the 5-year LC was 96% and the 10-year LC was 94%. Fig 2 shows that fraction size was correlated to local control for this histology.

**Fig 3:**
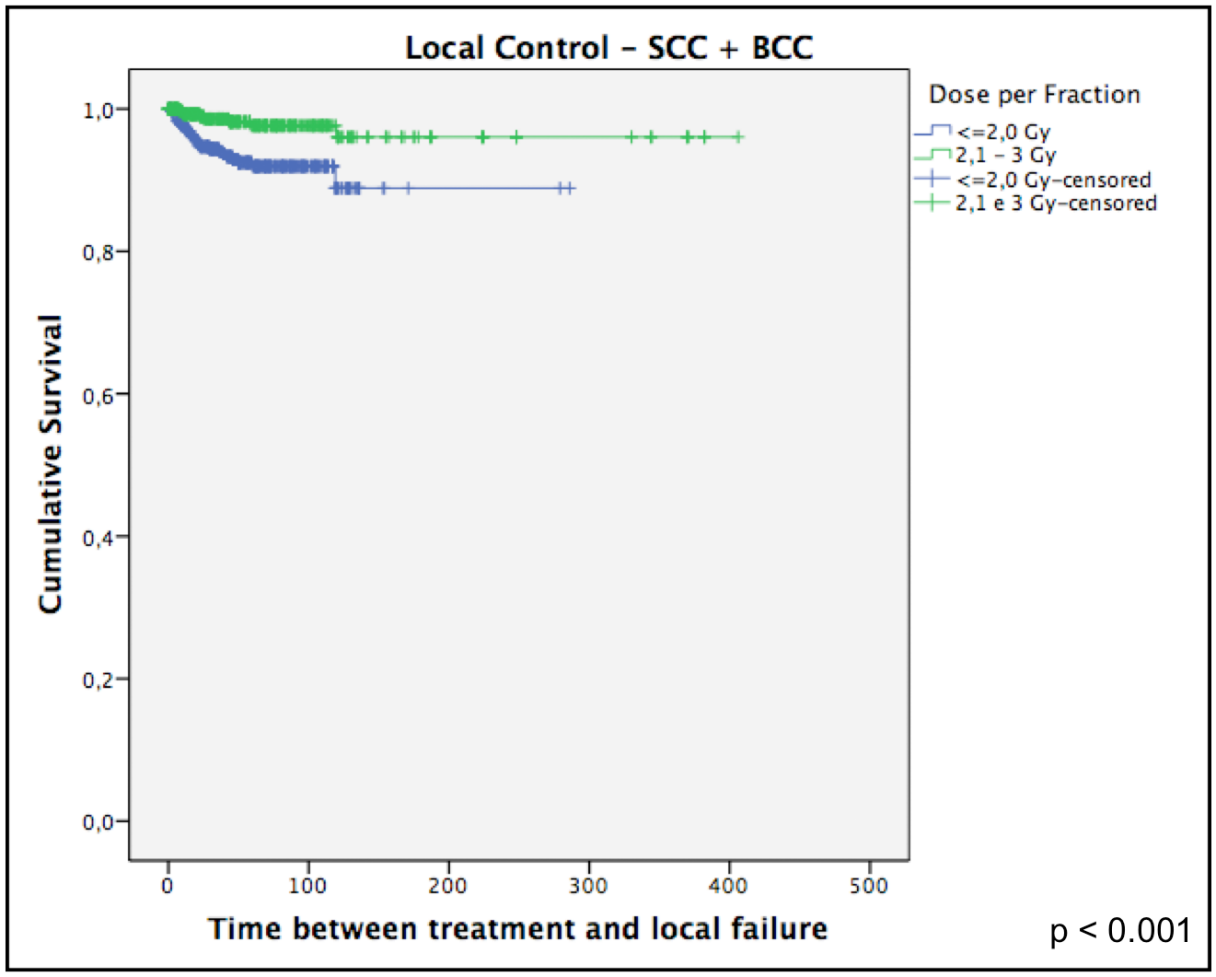
Local control only for SCC.

For SCC only, the corresponding rates of local control were 92% and 87%, respectively (p = 0.03). Fig 3 shows that dose per fraction was correlated to LC.

The model for logistic regression in the multivariate analysis was constructed with the following variables: T stage, dose per fraction and histology. The use of >2.0 Gy daily was identified as a significant prognosticator for improved LC on multivariate analysis (HR: 0.17; CI95%: 0.05–0.59).

Among the 720 cases with evaluable acute skin toxicity, only 64 were grade 3+ in severity. No significant difference was observed among the different prescribed fractionation schemes (p = 0.78) in terms of toxicity.

Table 2 summarizes information regarding tumor control and toxicity for different dose/fraction for the primary/adjuvant group.

**Table 2:** Tumor Control and Toxicity Between Different Fraction Sizes.

	All	Fraction size 2 Gy	Fraction size > 2 Gy	p-value
**RTOG G3/4 Toxicity Score (720 available)**	64	32	32	0,78
**Failures**	Local: 36	Local: 28	Local: 8	0,04*
	Regional: 4 (all SCC)	Regional: 3	Regional: 1	-
	Distant: 1 (SCC)	Distant: 1	-	-
**Local Failures per Histology**	BCC: 23	BCC: 16	BCC: 7	0.04
	SCC: 13	SCC: 12	SCC: 1	0.04
**Local Failures per Site**	Eyelid: 7 (all inferior)	Eyelid: 1	Eyelid: 6	-
	Nose: 6	Nose: 3	Nose: 3	-
	Pinna: 6	-	Pinna: 6	-
	Forehead: 2	Forehead: 2	-	-
	Internal canthus: 3	-	Internal canthus: 3	-
	Lips: 1 (inferior)	Lips: 1 (inferior)	-	-
	Face: 11	Face: 6	Face: 5	-
**Regional Failures per Site (all SCC)**	Pinna: 1	Pinna: 1	-	-
	Face: 3	Face: 2	Face: 1	-
**Distant Failures per Site (all SCC)**	Face: 1	Face: 1	-	-

*Local Control analysis only; regional and distant failures small number didn’t allow performing statistical comparisons between the groups

## Discussion

The use of SXRT has declined in recent years for several reasons, despite the satisfactory therapeutic results [10] with a corresponding increase in electron beam RT. In our center, kilovoltage radiation therapy is still widely used in clinical practice. The number of treated patients remains constant over the years.

Of all lesions analyzed for LR (invasive ones), there were 37 Local Recurrences what represents a raw recurrence rate of 4%. Among this 37 local recurrences, the treatment intention was primary radical in 35, adjuvant in one and salvage in another. This number may be overestimated, since we considered all lesions overlapping within the field of previous treatment, of which some may have been new lesions. Also we have to point that among these lesions that presented local recurrence only 1 was found to have high risk features: it was a T3 lesion in the internal eye canthus. We didn’t perform a separate analysis of the high risk lesions because of the reduced number of events.

Our data demonstrates relative success of SXRT as an adjuvant / salvage modality after previous surgery (only one failure among 17 treatments).

When analyzing the data available in the literature, we found results of tumor control very similar between the main series, as we can see below.

In 2005, Schulte KW et al [11] reported the results of 1267 lesions in 1113 patients treated by superficial x-ray therapy using higher than conventional total doses (based on tumor response during treatment). The total doses reached values greater than 80 Gy in some cases. They found a recurrence rate of 5%, not better than other descriptions.

In 2012, Cognetta AB et al [12] showed results of SXRT in 1715 lesions (712 BCC, 994 SCC and 9 with features of both components). When analyzing only SCC and excluding *in situ* lesions, the 2 and 5-year LF rates were 1% and 7%, respectively. They also found a 2 and 5-year LF rate of 2% and 5% for the *in situ* lesions. In total, from the 1715 lesions, 45 were considered to be recurrent, what represents a 2-year and 5-year LF of 2% and 5%, respectively, for all tumors. All recurrences were amenable to salvage with surgery. There was no regional or systemic progression.

Our results are comparable to these and other series [13–20] as can be seen in the Table 3.

**Table 3:** Other Series of SXRT.

Author	Lesions	Site	Energy (KeV)	Daily dose (Gy)	Total dose (Gy)	Result
**Schulte (2005)** [11]	1267	H&N	10–100	5	< 80	Recurrence rate: 5.1% (all) 4.5% (BCC); 6.9% (SCC)
**Cognetta (2012)** [12]	1715	H&N	Most 80	5–7	35	5y cumulative recurrence-rate: 5% (all); 4.2% (BCC); 5.8% (SCC)
**Caccialanza (2003)** [13]	405	Nose	55–120	2–5	40–85	5y Cure: 88.6%
**Locke (2001)** [14]	317	Whole body	-	<2–> 4	<40–>60	Overall LC: 94%
**Ashby AA (1989)** [15]	454	Whole body	-	Median 4	6–48	5y LC: 90%
**Zagrodinik (2003)** [16]	175	Whole body	20–50	2–8	40–60	5y Overall Recurrence rate (BCC only): 15.8%
**Caccialanza (2005)** [17]	108	Pinna	55–120	2,5–5	45–70	5-y cure-rate: 78% (BCC+SCC)
**Hernández-Machin (2007)** [18]	710	Whole body	14–50	4–9	36–55	5-y cure-rate: 94.5% (BCC); 92.7% (SCC)
**Caccialanza (2009)** [19]	671	Nose	55–60	5	30–75	5y cure-rate: 88.09% (all)
**Silva (2000)** [20]	278	Pinna	100–250	2–20	35–65	5y LC: 79.2% (all); 83% (BCC); 79% (SCC)
**Current Serie**	1021	H&N	60–200	2–2,5	50–60	5y LC: 95.6% (BCC); 91.9% (SCC)

In the study of Cognetta et al [12], the median time interval until recurrence was 34.7 months. In our study, the median FU is 44 months, what means that most recurrences were detected.

Regarding our findings of improved outcomes with hypofractionation, there are consistencies in the literature. In 2001, Locke et al [14] reviewed patterns of failure and outcomes of 531 lesions in the whole body of 468 patients (80% performed SXRT isolated or in combination with electron beam). The FU was 5,8 years. The overall local control was 89% (93% for untreated and 80% for recurrent lesions). When looking only at those treated with SXRT, overall LC was 94%. When dividing by histology, the overall local control was 92% for BCC and 80% for SCC. On multivariate analysis, local failure was related to the daily dose fractionation, maximal diameter and tumor type. BCC 1.1–5 cm showed a trend for better local control as the fraction size increased from <2 to 3–4 Gy.

A practical limitation of this study is that we were unable to identify the aggressiveness of the lesions, so selection bias can be present. In general, our institution uses to treat more aggressive lesions with surgery or combined therapy, although some of these lesions could have entered this series. There are some reports in the literature showing reasonable control for aggressive BCC using radiation therapy [21–23], albeit still worse than the results achieved in non-aggressive ones.

Another factor that has to be considered is that the patients treated by this Oncological Hospital are, as a rule, from poor regions or rural areas. This feature alone may be related to sun exposure without proper protection. Moreover, it is unusual to use Personal Protective Equipment (PPE) for protection against the risks of the job (such as exposure to pesticides); even when used, this does not rule out the incorrect use of PPE by the lack of control of public departments and the socio-cultural characteristics of this population.

Besides, on admission of patients in our hospital it goes through review of the surgeon and the radiation oncologist; the choice of treatment is based on multidisciplinary decision and assessment of curative and cosmetic effects of different treatments. This can cause a selection bias inherent in the study design.

When looking at the surgical studies, our results are comparable to those described in Mohs surgeries series [24–26].

## Conclusion

This study suggests that SXRT is a reasonable treatment of N-MSC and should be considered as an option when deciding therapy.

SXRT for non-melanoma skin cancer of the head and neck is well tolerated, achieves excellent local control, and should continue to be recommended in the management of this disease.

Fractionation schedules using >2.0 Gy daily appear to be associated with improved LC for both SCC and BCC.
